# Implementation of a Telemental Health Training Program Across a Mental Health Department

**DOI:** 10.1089/tmr.2020.0011

**Published:** 2021-01-21

**Authors:** Bradford L. Felker, Meghan M. McGinn, Erika M. Shearer, Gina T. Raza, Sari D. Gold, Jean M. Kim, Sasha M. Rojas, Milena S. Roussev, Ruth L. Varkovitzky, Huiting Liu, Kate L. Morrison, Russell A. McCann

**Affiliations:** ^1^ Department of Veterans Affairs VA Puget Sound Health Care System, Seattle, Washington, USA.; ^2^ University of Washington Department of Psychiatry and Behavioral Sciences, Seattle Washington, USA.; ^3^ Department of Veterans Affairs VA Puget Sound Health Care System, Tacoma, Washington, USA.

**Keywords:** telemental health, implementation science, training program

## Abstract

**Introduction:** Telemental health (TMH) has increased substantially. However, health care systems have found it challenging to implement TMH ubiquitously. A quality improvement project guided by implementation science methodology was used to design and implement a TMH training program.

**Materials and Methods:** Implementation science methodology (Promoting Access to Research Implementation in Health Services, Reach-Effectiveness-Adoption-Implementation-Maintenance, Implementation/Facilitation) provided the framework to design and implement the training program. A total of 100 interdisciplinary mental health providers from outpatient mental health clinics participated.

**Results:** Providers reported satisfaction with the training program. Results indicated that the training increased providers' TMH knowledge and competence. The number of providers using TMH and patients who received TMH nearly doubled.

**Conclusions:** Implementation science methodology was important in creating an organizational framework at this facility to design, evaluate, and implement an innovative TMH training program.

## Introduction

In a recent survey, 22% of adults in the United States with mental illness were denied access to necessary mental health services.^1^ Approximately 34% of U.S. counties have no psychologists,^2^ and nearly 54% have no psychiatrists.^3^ Telemental health (TMH) via synchronous video-based technology has great potential to improve access to quality care^4,5^; however, large-scale TMH adoption through which every provider in a health care system is using this modality remained elusive in the pre-COVID-19 era.^6^

The challenge in achieving broad TMH utilization was likewise observed in the VA Puget Sound Health Care System (VAPSHCS) where, despite TMH training efforts, adoption of TMH among providers remained relatively low.^7^ Given that TMH utilization is significantly correlated with quality training combined with implementation efforts,^4^ this quality improvement project (QIP) aimed to implement a more comprehensive TMH training program guided by the Implementation Science methodology literature. The Promoting Access to Research Implementation in Health Services (PARiHS) framework was chosen to guide this QIP due to the flexibility in application and emphasis on identifying and implementing critical elements (evidence, context, facilitation) that provide structure to a project.^8,9^ To identify clear outcome variables that were comprehensive and relevant, the Reach-Effectiveness-Adoption-Implementation-Maintenance (RE-AIM) methodology was chosen.^10,11^ Finally, to guide the actual process of implementation of this training program, the Evidence-Based Quality Improvement Implementation/Facilitation methodology was used.^12^

This QIP resulted in the creation of a novel TMH training program was implemented between 2017 to 2019. The primary aim of this article is to describe the development, implementation, and evaluation of this TMH training program. As this QIP concluded before the massive shift to virtual care that occurred as part of the response to the COVID-19 pandemic, a secondary aim of this article is to review the extent to which the VA health care system providers have adopted TMH as a modality in the post-COVID-19 era, and consider whether a formal TMH training program such as the one developed as part of this QIP remains relevant.

## Materials and Methods

The work presented herein was reviewed jointly by the VAPSHCS Human Research Protection Program and by the Office of Transformation, Quality, Safety & Value Service Line and was determined not to constitute human subjects research. It met all criteria necessary to be designated as an approved QIP.

Staff from the VAPSHCS TMH service met for a 2-day retreat to inform this mixed-methods QIP, which culminated in the development and implementation of a novel TMH training program that was designed to encourage system-wide TMH adoption. During this retreat, staff critically reviewed the previous TMH training project and determined that the training program lacked structure, clarity (especially when communicating goals with leadership), coordination, and collaboration with teams where the training would take place, ability to provide detailed training, clear goals described in outcome measures, and ongoing support of trainees. The staff looked to the training literature and Implementation Science literature. The staff determined that a subsequent training program would benefit from the structure provided by PARiHS, clearly defined outcome measures detailed by RE-AIM, and a process that guided the training program's actual implementation, which is described with Evidence-based Quality Improvement Implementation/Facilitation.^8–12^

Core elements of the PARiHS methodology include the following: *Evidence, Context, and Facilitation.* The *Evidence* included information from the literature on other system-wide TMH training efforts, experience from TMH service clinicians, acceptance by Veterans, and knowledge of the Mental Health Service Line based on previous training efforts. Regarding *Context*, leadership support was deemed necessary. The information gathered in the *Evidence* element was incorporated into the training program and presented to senior leadership when seeking approval for this project. In addition, within *Context*, the culture of those clinics identified for implementation was considered. Within these clinics, “clinical champions” (e.g., early adopters within each clinical team) were identified to help provide training and support within each clinic. For this project, the RE-AIM evaluation methodology^10,11^ was used and included the following outcome measures:

- *Reach* measured the number and types of providers trained.
- *Effectiveness* captured the number of individual Veterans receiving TMH services.
- *Adoption* included number of providers who completed the training and who completed at least one TMH encounter following the training.
- *Implementation* was assessed via a leadership-approved pre- and post-training self-report assessment questionnaire, which was administered to track change in knowledge, skills, and interest in delivering TMH. Perceptions of barriers to the use of TMH and satisfaction with the training experience were also evaluated.
- *Maintenance* outcomes were assessed at 3, 6, and 12 months post-training and included participants' perceived TMH knowledge, skills, and interest. The utilization of TMH by providers and patients was also evaluated over time.

The *Facilitation* element of PARiHS was guided by the Evidence-Based QI Implementation and Facilitation literature.^12^ The TMH service providers were considered *External Facilitators* and served as the subject matter experts to implement the training. They were paired with clinical champions (early adopters) and team leads for the teams targeted to receive the training and identified as *Internal Facilitators*. *Internal Facilitators* from each team provided consultation to the *External Facilitators* regarding the unique clinical and cultural aspects of their team (e.g., patients served, types of services provided, administrative needs, technological needs). *External* and *Internal Facilitators* tailored the TMH training program to address clinic-specific culture and barriers and meet unique clinic goals.

The TMH training program was developed and implemented with support from the VAPSHCS Mental Health Service Line leadership team. The TMH training program was structured as follows. Participants: (1) completed two VA-required online VA TMH training courses; (2) attended an 8-hour workshop specific to the practical aspects of providing TMH (e.g., determining patient appropriateness, safety planning, billing, documentation, prescribing), hands-on training and practice using the TMH equipment, and a VA-required TMH skills assessment; and (3) upon completion, were encouraged to engage in TMH with at least two patients and attend at least 10, 1-hour TMH consultation calls where they could ask questions related to TMH clinical or implementation issues. The TMH trainings were offered on a recurring basis. A total of 16 TMH trainings were facilitated. This project took place between 2017 and 2019.

## Results

### Reach outcomes

In the 2-year period following initiation of the TMH training program (2017–2019), 100 providers participated in the TMH training program (72 staff and 28 trainees who operate under licensed staff). The participants' disciplines included psychology (37%), social work (22%), other/not specified (19%), psychiatry (17%), and nursing (5%). The clinics participating included outpatient mental health (49%), undisclosed (26%), outpatient addictions treatment (13%), inpatient mental health (4%), primary care mental health (4%), and other (4%; e.g., trainee rotating through multiple clinics).

### Effectiveness outcomes

The number of Veterans who received TMH increased from 1301 (5% of Veterans who received mental health) to 2755 (10%) during the 2-year period following training initiation (2017–2019). Of the 2755 Veterans who received TMH post-training, 449 (16%) received services from providers who participated in the training.

### Adoption outcomes

The percentage of providers with at least one TMH visit increased from 16% to 39%, comparing the 2-year period before the training with the 2 years after. The number of TMH encounters increased from 6752 to 14,124 in the 2-year period following training initiation.

### Implementation outcomes

#### Full sample

Following the training, 95% of providers agreed (*n* = 42) or strongly agreed (*n* = 35) that they were satisfied with the training provided. In addition, 95% of providers agreed (*n* = 50) or strongly agreed (*n* = 28) that the amount of information covered was enough to begin using TMH. After completion of the training, 76% of participants agreed (*n* = 45) or strongly agreed (*n* = 17) that they felt confident using TMH. A Wilcoxon signed-rank test was used to determine significant differences in providers' perception of knowledge, skills, and interest in using TMH from pre- to post-training.

#### Training participants with no prior TMH experience

Among participants with no previous TMH experience, the most frequently endorsed barrier to using TMH *pretraining* was administrative burden (28%), followed by preference for in-person appointments (25%), not having completed the training (25%), concern about increased workload (17%), some other specified reason (7%; e.g., finding a space with technology capability, lack of facility support, lack of technological skills, technological problems), lack of supervisor support (4%), lack of patient interest (4%), and lack of provider interest (2%). *Post-training* for these participants, the most frequently endorsed barrier was lack of patient interest (45%), followed by administrative burden (20%), preference for in-person appointments (18%), concern about increased workload (11%), not having completed all of the training components (6%), lack of supervisor support (4%), lack of provider interest (4%), and some other reason (4%).

#### Training participants with prior TMH experience

Among participants with previous TMH experience, the barriers to using TMH identified *pretraining* included administrative burden (13%), preference for in-person appointments (5%), some other reason (2%), lack of supervisor support (1%), lack of patient interest (1%), and concern that in-person clinic grids might appear underutilized (1%). The *post-training* barriers reported by these participants were lack of supervisor support (8%), concern about increased workload (4%), administrative burden (4%), lack of patient interest (2%), concern about TMH cases making in-person clinic grids appear underutilized (2%), some other specified reason (2%; e.g., technical support, telehealth being more useful for reoccurring sessions), and preference for in-person appointments (1%).

Results indicated that providers' perceptions of knowledge (*Z* = −6.67, *p* < 0.001), skills (*Z* = −6.09, *p* < 0.001), and interest (*Z* = −2.54, *p* = 0.01) in using TMH each significantly increased after the training.

### Maintenance outcomes

Wilcoxon signed-rank tests compared post-training responses through 3-, 6-, and 12-month follow-up assessments. Results showed no differences in providers' perceived knowledge, skills, or interest over time, indicating maintenance of gains.

## Discussion

Despite substantial evidence in support of TMH, providers' adoption of TMH had been slow before the COVID-19 pandemic in early 2020.^13–16^ Poor adoption rates occurred despite significant investments and policy changes that encouraged use.^13,17^ Successful factors identified to improve telehealth adoption rates include choice of applications, clinician engagement, business practices, technology, training, and use of sustained evaluation.^18^ However, it had not been clear how best to incorporate such factors into a successful training program.

The QIP described in this article used implementation science methodology to develop and implement an innovative TMH training program aimed to overcome previous organizational barriers to widespread TMH use. PARiHS criteria provided a framework to organize the program and communicate to stakeholders.^8,9^ Evaluation measures were organized using RE-AIM criteria,^10,11^ and the implementation was guided by the Evidence-Based Quality Improvement Implementation/Facilitation literature, via the use of *External* and *Internal Facilitators*.^12^ The TMH training program was provided to 100 interdisciplinary clinicians throughout the Mental Health Service Line of a large VA health care system over the course of 2 years.

Overall, most providers were satisfied with the TMH training and reported a post-training increase in TMH knowledge, skills, and interest. These gains were maintained across 12-month follow-up. This structured TMH training program coincided with an approximately twofold increase in the number of clinicians and Veterans using TMH services in the VAPSHCS. It is hypothesized that the evidence-based Implementation Science methodology provided structure and clear guidance on how to obtain leadership support, where to target the intervention, how to organize relevant comprehensive outcome measures, and offered a process that facilitated the implementation by increasing coordination and collaboration with the teams targeted for the intervention; all contributing to the success of this training program. In addition, we provided extensive hands-on training and prolonged support so that the participants had time to develop experience and competence.

This TMH training program effort preceded the COVID-19 pandemic. When COVID-19 began, the need for rapid adoption of TMH increased. However, health care systems varied in their ability to implement TMH in response to the pandemic. In June 2020, the average VA health care system provided home-based TMH to 26.3% of Veterans who might be expected to want such a service, based on the number of Veterans who received outpatient mental health services in-person in June of the previous year. The VAPSHCS home-based TMH adoption rate during the same time frame was observed at 48.5%, ranking seventh highest out of 140 VA health care systems.

The COVID-19 pandemic certainly had a major impact on expanding TMH, but the pandemic alone did not result in health care systems adopting TMH equally. This project was completed before COVID-19, and the pandemic itself caused a massive increase in TMH utilization. This raises a question as to whether structured TMH training in a post-COVID era is still needed to facilitate staff adoption of this treatment modality. The observed variability in how extensively VA health care systems adopted TMH after the start of the COVID-19 pandemic, considered with VAPSHCS' notably above-average TMH utilization, seems to suggest that, for this facility, having a structured TMH training program was important. Notably, there were several TMH pilot projects that took place at local and regional VAs before this training program. These earlier TMH training efforts likely helped foster staff interest and experience using TMH, and in doing so contributed to the relatively strong TMH adoption rate observed in this health care system during the early months of the COVID-19 pandemic.

Limitations of this QIP include a lack of control group to compare TMH implementation without training. However, TMH utilization after this training program was robust, providing support that such a training effort contributed to this success. One interesting result was that participants with no previous TMH experience compared with those participants with previous TMH experience reported that lack of patient interest was a frequently endorsed barrier post-training (45% compared with 2% by those with previous TMH experience). It is possible that this finding highlights remaining biases that participants who had no prior TMH experience held regarding patient interest in telehealth, despite the literature indicating that patients are often more willing to use TMH than providers.^19–21^ Future examinations of staff use of TMH may benefit from examining staff perceptions of patient interest after engaging in TMH. Finally, as this TMH training program was completed in a single large federal health care system, results may not be generalizable.

## Conclusions

The use of established implementation science methodologies provided a structural framework to implement a TMH training program that facilitated staff training and skill development at our institution. Overall, the training was well received by providers and led to an increase in TMH use by both patients and staff. In addition, when considering the variability observed in TMH adoption rates among VA health care systems in the post-COVID era with the above-average TMH adoption at the site where this TMH training program was implemented, it seems to suggest that having a structured TMH training program might remain relevant, and that the pandemic alone has not yet resulted in universal provider adoption of TMH. An unreviewed preprint version of this material was posted by Research Square in Europe PMC.^22^

## Availability of Data and Materials

The data sets generated and/or analyzed during the current study are not publicly available (stored on secure VA databases that require official VA approval for access). Some data are available from the corresponding author on reasonable request. All training materials are available upon request.

## Abbreviations Used

HBTMH: home-based TMH
PARiHS: Promoting Access to Research Implementation in Health Services
QI: quality improvement
QIP: quality improvement project
RE-AIM: Reach-Effectiveness-Adoption-Implementation-Maintenance
TMH: telemental health
VAPSHCS: VA Puget Sound Health Care System

## References

[1] Mental Health America. The state of mental health in America 2020. 2020. Available at https://mhanational.org/sites/default/files/State%20of%20Mental%20Health%20in%20America%20-%202020.pdf Accessed 61, 2020

[2] Lin L, Stamm K, Christidis P County-level analysis of U.S. licensed psychologists and health indicators. 2016. Available at https://www.apa.org/workforce/publications/15-county-analysis/report.pdf Accessed 61, 2020

[3] Beck AJ, Page C, Buche J, et al. Estimating the distribution of the U.S. psychiatric subspecialist workforce. 2019. Available at www.behavioralhealthworkforce.org/wp-content/uploads/2019/02/Y3-FA2-P2-Psych-Sub_Full-Report-FINAL2.19.2019.pdf Accessed 61, 2019

[4] Gibson K, O'Donnell S, Coulson H, et al. Mental health professional's perspectives of telemental health with remote and rural First Nations communities. J Telemed Telecare 2011;17:263–267 21824967 10.1258/jtt.2011.101011

[5] Pruitt LD, Luxton DD, Shore P Additional clinical benefits of home-based telemental health treatments. Prof Psychol Res Pract 2014;45:340–346

[6] Ellimoottil C, An L, Moyer M, et al. Challenges and opportunities faced by large health systems implementing telehealth. Health Affair 2018;37:1955–1959 10.1377/hlthaff.2018.05099 30633667

[7] Perry K, Gold S, Shearer EM Identifying and addressing mental health providers' perceived barriers to clinical video telehealth utilization. J Clin Psychol 2019 DOI: 10.1037/a0035461 30859573

[8] Stetler CB, Damschroder LJ, Christian D, et al. A guide for applying a revised version of the PARIHS framework for implementation. Implementation Sci 2011;6:99 10.1186/1748-5908-6-99 PMC3184083 21878092

[9] Kitson AL, Rycroft-Malone J, Harvey G, et al. Evaluating the successful implementation of evidence into practice using the PARiHS framework: theoretical and practical challenges. Implementation Sci 2008 DOI: 10.1186/1748-5908-3-1 PMC2235887 18179688

[10] Gaglio B, Shoup JA, Glasgow RE The RE-AIM framework: a systematic review of use over time. Am J Public Health 2013;103:e38–e46 10.2105/AJPH.2013.301299 PMC3698732 23597377

[11] Glasgow RE, McKAy HG, Piette JD, et al. The RE-AIM framework for evaluating interventions: what can it tell us about approaches to chronic illness management? Patient Educ Couns 2001;44:119–127 11479052 10.1016/s0738-3991(00)00186-5

[12] Kirchner JE, Ritchie MJ, Pitcock JA, et al. Outcomes of a partnered facilitation strategy to implement primary care-mental health. Gen Intern Med 2014 DOI: 10.1007/s11606-014-3027-2 PMC4239280 25355087

[13] Broens TH, Vollenbroek-Hutten MM, Hermens HJ, et al. Determinants of successful telemedicine implementations: a literature study. J Telemed Telecare 2007;13:303–309 17785027 10.1258/135763307781644951

[14] Edirippulige S, Armfield NR Education and training to support the use of clinical telehealth: a review of the literature. J Telemed Telecare 2017;23:273–282 26892005 10.1177/1357633X16632968

[15] Handler TJ Hype cycle for telemedicine. Gart Ind Res 2007;G00233474:1–56

[16] van Dyk L A review of telehealth service implementation frameworks. Int J Environ Res Public Health 2014;11:1279–1298 24464237 10.3390/ijerph110201279 PMC3945538

[17] Zanaboni P, Wootton R Adoption of telemedicine: from pilot stage to routine delivery. BMC Med Inform Decis Mak 2012;12:1 22217121 10.1186/1472-6947-12-1 PMC3280930

[18] Yellowlees P Successfully developing a telemedicine system. J Telemed Telecare 2005;11:331–336 16238833 10.1258/135763305774472024

[19] Polinski JM, Barker T, Gagliano N, et al. Patients' satisfaction with and preference for telehealth visits. J Gen Intern Med 2016;31:269–275 26269131 10.1007/s11606-015-3489-x PMC4762824

[20] Monthuy-Blanc J, Bouchard S, Maiano C, et al. Factors influencing mental health providers' intention to use telepsychotherapy in First Nations communities. Transcult Psychiatry 2013;50:323–343 23666941 10.1177/1363461513487665

[21] Simms DC, Gibson K, O'Donnell S To use or not to use: clinicians' perceptions of telemental health. Can Psychol 2011;52:41–51

[22] Felker BL, McGinn MM, Shearer EM, et al. Implementation of a telemental health training program across a mental health department. Preprint from Research Square 2020 DOI: 10.21203/rs.3.rs-51846/v2 PMC7869919 33575684

